# Automatic quantitative analysis of experimental primary and secondary retinal neurodegeneration: implications for optic neuropathies

**DOI:** 10.1038/cddiscovery.2016.31

**Published:** 2016-05-23

**Authors:** B M Davis, L Guo, J Brenton, L Langley, E M Normando, M F Cordeiro

**Affiliations:** 1 Glaucoma and Retinal Neurodegeneration Research, Visual Neuroscience, UCL Institute of Ophthalmology, Bath Street, London EC1V 9EL, UK; 2 Western Eye Hospital, Imperial College Healthcare Trust, London, UK

## Abstract

Secondary neurodegeneration is thought to play an important role in the pathology of neurodegenerative disease, which potential therapies may target. However, the quantitative assessment of the degree of secondary neurodegeneration is difficult. The present study describes a novel algorithm from which estimates of primary and secondary degeneration are computed using well-established rodent models of partial optic nerve transection (pONT) and ocular hypertension (OHT). Brn3-labelled retinal ganglion cells (RGCs) were identified in whole-retinal mounts from which RGC density, nearest neighbour distances and regularity indices were determined. The spatial distribution and rate of RGC loss were assessed and the percentage of primary and secondary degeneration in each non-overlapping segment was calculated. Mean RGC number (82 592±681) and RGC density (1695±23.3 RGC/mm^2^) in naïve eyes were comparable with previous studies, with an average decline in RGC density of 71±17 and 23±5% over the time course of pONT and OHT models, respectively. Spatial analysis revealed greatest RGC loss in the superior and central retina in pONT, but significant RGC loss in the inferior retina from 3 days post model induction. In comparison, there was no significant difference between superior and inferior retina after OHT induction, and RGC loss occurred mainly along the superior/inferior axis (~30%) *versus* the nasal–temporal axis (~15%). Intriguingly, a significant loss of RGCs was also observed in contralateral eyes in experimental OHT. In conclusion, a novel algorithm to automatically segment Brn3a-labelled retinal whole-mounts into non-overlapping segments is described, which enables automated spatial and temporal segmentation of RGCs, revealing heterogeneity in the spatial distribution of primary and secondary degenerative processes. This method provides an attractive means to rapidly determine the efficacy of neuroprotective therapies with implications for any neurodegenerative disorder affecting the retina.

## Introduction

Secondary neurodegeneration is reported to play a significant role in many diseases of growing socioeconomic and health-economic concern, including neurodegenerative diseases, spinal cord injuries and optic neuropathies.^1–7^ It is thought to occur through a combination of excitotoxicity, inflammation and oxidative stress pathways, which provide attractive therapeutic targets as secondary neurodegeneration is clinically more modifiable than primary.^8–10^ Although substantial progress has been made in our understanding of these disorders in recent years, including recognition of similarities between these conditions,^11,12^ there are presently few effective disease-modifying therapies to slow or reverse the course of neurodegeneration.

One of the most common ophthalmic neurodegenerative diseases is glaucoma, which comprises a distinctive group of progressive optic neuropathies and is the leading cause of irreversible blindness globally.^13^ Gradual degeneration of RGCs and optic nerve damage, with characteristic changes in appearance of the optic disc, is responsible for the progressive visual field loss. Classically (but not exclusively), this manifests as an initial permanent loss of peripheral vision, leading to central vision defects as the disease progresses.^14,15^ Presently, there is no curative treatment for glaucoma, with existing therapies targeting raised intraocular pressure (IOP), the major and modifiable risk factor.^16,17^ Glaucoma is a complex and multifactorial disease, with variation in disease progression, and vision loss continuing to occur in some patients, despite well-controlled IOPs,^18^ suggesting that secondary RGC degeneration plays an important role in glaucoma pathology.^10,19^

Rodent models of glaucoma have proven essential in understanding disease progression and assessing efficacy of therapeutic interventions. In this respect, quantitative assessment of RGC loss is a commonly used end point to assess experimental glaucomatous degeneration and therapeutic intervention efficacy.^10,20^ Although none are exact models of human disease, they allow study of different mechanistic aspects.^21^ Partial optic nerve transection (pONT) is a useful model first described in cynomolgus monkeys and Wistar rats.^7,22^ This model facilitates segregation of primary and secondary degeneration processes by transecting only the superior optic nerve, enabling mechanisms of RGC degeneration and efficacy of therapeutic interventions to be assessed.^10,23,24^ Another, well-established rodent model of glaucoma is the Morrison model of ocular hypertension (OHT), which involves injection of hypertonic saline into the episcleral veins to induce elevated IOP via induction of sclerosis of the trabecular meshwork impeding aqueous drainage.^25^ The resulting elevated IOP is correlated with increased RGC apoptosis^26,27^ and functional assessment of RGC loss suggests moderate sustained elevation of IOP results in peripheral vision loss.^28^

**Figure 1:**
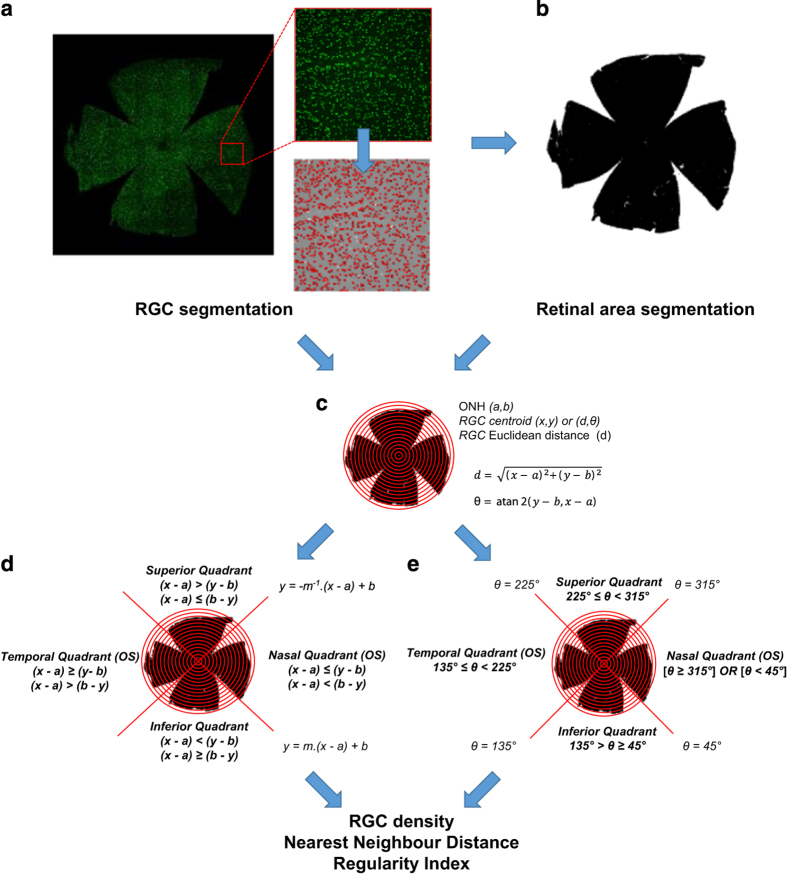
Diagram of RGC segmentation and counting used in this study. (**a**) Identification of RGC number, area and centroid from Brn3a-labelled retinal whole-mounts was completed using previously described methods.^10^ (**b**) Retinal area was determined by first creating a binary mask of the whole-mount area before applying a customised ImageJ script to measure the retinal area (black pixels) in each retinal segment (ring and quadrants). (**c**) On defining the centre of the ONH, a series of 15 concentric rings with 0.3 mm radii were drawn and the position of each RGC determined by calculating the Euclidean distance [*D*] of each RGC relative to the ONH using ring boundaries as thresholds. RGCs were further segmented into quadrants (**d **and **e**) (where *m*=*b*/a) defined using Cartesian (**d**) or polar coordinates (**e**) which gave identical results. For each segment, in each case RGC density, average nearest neighbour distance and regularity index were calculated as described in the text.

Brn3a is a POU-domain transcription factor considered a good histological RGC marker, due to its high RGC specificity and good agreement with retrograde axonal tracer Fluoro-Gold (97% RGC co-labelling in rats).^29^ As Brn3a is localised to RGC nuclei, immunohistochemistry of retinal whole-mounts yields images from which RGCs can be readily segmented (Figure 1a). Quantification of Brn3a RGCs is frequently obtained by manually counting retinal segments;^7,30–33^ yet a limitation of this approach is the high variability in RGC density between central and peripheral rodent retina, which can complicate RGC quantification by sampling.^34^ To address these concerns, whole retinas need to be assessed and several groups, including our own, have developed tools to automatically count Brn3a-labelled RGCs, results of which are most frequently presented quantitatively as average whole-mount RGC density (RGC/mm^2^) or qualitatively as isodensity maps.^10,29,35,36^

In this study we describe a new segmentation algorithm for automatic determination of regional RGC density, nearest neighbour distance (NND) and regularity index (RI), using a series of 15 concentric rings centred on the optic nerve head (ONH), segmented into quadrants. We apply this method to investigate the patterns and natural history of RGC loss in two well-established models of glaucoma: the Morrison’s model of OHT^25^ and pONT,^23^ to assess primary and secondary neuronal degenerative processes as potential therapeutic targets.

## Results

### Establishment of a novel algorithm for whole-retinal RGC analysis and spatial segmentation

Using the previously established automated RGC counting algorithm,^10^ a method was established to spatially segment Brn3a-labelled retinal whole-mounts. This enabled the spatial and temporal distribution of RGC loss to be automatically determined (Figure 1). This script was initially used to evaluate a series of control eyes: average retinal area (52.4 ±5.1 *μ*m), Brn3a positive RGC number (82 592±681 RGCs/retina) and average whole-retinal RGC density (1695±23.3 RGC/mm^2^) were documented.

Established retinal neuronal mosaic parameters include NNDs and Regularity Indices (RIs). NND refers to the distance between each neuron and its nearest neighbour in the mosaic, presented as the mean NND.^37^ RI presents one of the most popular methods for quantifying retinal mosaics and comprises a spatial statistic derived from the frequency distribution of NNDs, and is calculated by dividing the mean NND by the standard deviation of the NDD of a population within a sample field (equation (1)).^38^ As orderly retinal mosaics possess an approximate Gaussian NND frequency distribution and random retinal mosaics possess a Poisson distribution, greater RI ratios will be observed for Gaussian (ordered) distributions of retinal cells.^39^ Typically, the RI for neurons arranged randomly is 1.9, and the more regular the arrangement, the higher the RI.^37^ The RI for retinal mosaics observed to date, is reported to be between 3 and 8.^38^ However, the exact threshold for determining whether the mosaic is non-randomly arranged depends on the number of neurons and the geometry of the field. In addition, the physical size of soma may introduce lower limits (an exclusion radius) onto NNDs which may adversely influence RI calculations.^37^ For control DA rat retina, an average RI of 2.86±0.03 was obtained, which is indicative of a retinal mosaic with a regular arrangement.^37^

### Application of the whole-retinal algorithm for the spatial and temporal assessment of the RGC mosaic in rodent models of optic neuropathy

**Figure 2:**
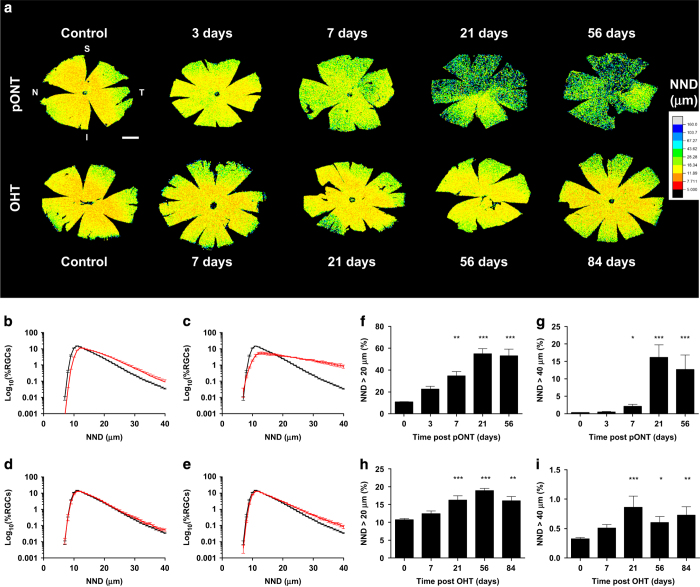
Change in NND distribution over the course of pONT and OHT models. (**a**) Colour maps comparing changes in RGC nearest neighbour distance in pONT and OHT models over time, scale bar =2 mm. (**b**–**e**) Longitudinal changes in global NND distribution over the course of the pONT (**b** and **c**) and OHT (**d** and **e**) models (red) *versus* naïve controls (black). (**f**–**i**) Graphs illustrating the proportion of the normalised NND distribution with NNDs (**b** and **c**) greater than 20 *μ*m (**f** and **h**) and 40 *μ*m (**g** and **i**) in the pONT (**f** and **g**) and OHT (**h** and **i**) models. Difference *versus* naïve controls determined using a one-way ANOVA with Dunns *post hoc* test.

To spatially segment retinal RGC populations, the centroid of each Brn3a-labelled RGC was next used to calculate its position relative to the ONH using vector coordinates, before a series of 15 concentric rings subdivided into four quadrants were constructed from which regional RGC density, NND and RI parameters were determined (Figures 1c–e). This RGC segmentation algorithm was applied to a total of 83 whole-mounted Brn3a-labelled retina 3, 7, 21 and 56 days after pONT induction or 7, 21, 56 and 84 days post OHT induction. A significant reduction in RGC density was observed from 3 days of pONT model induction and after 7 days of OHT model induction, and RGC density declined by 71±11 and 23±5% over the course of pONT and OHT models, respectively, compared with a population of naïve eyes (Table 1). Upon completion of RGC segmentation, NNDs were computed from each Brn3a positive RGC, and combined with RGC centroid data to produce qualitative NND colour maps (Figure 2a), where the smallest NNDs (red) represent regions of greatest RGC density, larger NNDs (green/blue) regions of low RGC density. Quantitative analysis of normalised NND distributions (Figures 2b–i) reveals that over the course of pONT and OHT models, overall NND distribution shifted to greater values, suggestive of diffuse RGC loss (primary degeneration).^40,41^ The concurrent increase in skew of the NND distribution to higher NND values (Figures 2b–e) *versus* naive controls is evidence of an additional component of clustered RGC loss, which may be indicative of secondary degenerative processes.^40,41^

### RGCs loss occurs predominantly in the superior-central retinal in the pONT model

**Figure 3:**
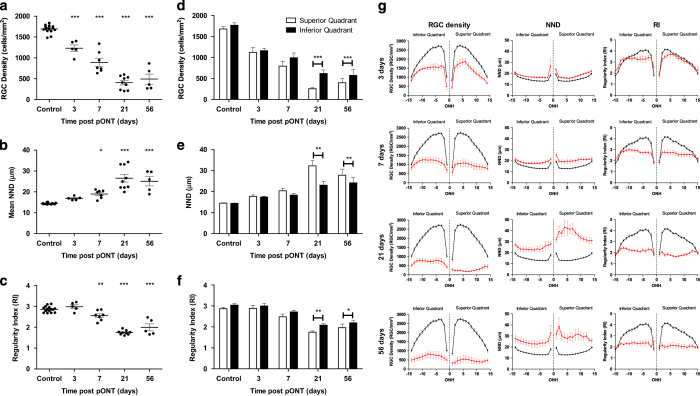
Retinal ganglion cell (RGC) survival after partial optic nerve transection (pONT) in the DA rat. Whole-retinal profiles of pONT induced loss of (**a**) RGC density, (**b**) nearest neighbour distance and (**c**) regularity index demonstrating significant reducing in RGC populations and retinal mosaic regularity over the course of this model (one-way ANOVA with Dunnets’ *post hoc* test *versus* naïve controls). (**d**–**f**) Comparison of RGC survival in the superior *versus* inferior retinal quadrants after pONT in the DA rat . (**d**) RGC density, (**e**) nearest neighbour distance and (**f**) regularity index in the superior (white) and inferior (black) retinal quadrants as outlined in Figure 1. RGC loss was significantly greater in the superior retina (paired two-tailed *T*-test) compared with the inferior retinal quadrant. (**g**) Further segmentation of RGC populations in the superior and inferior quadrants into a series of 15 non-overlapping concentric rings centred on the ONH suggest the greatest loss of RGC density occurs in the superior retinal quadrant *versus* the inferior quadrant over the course of the pONT model (red) when compared with naïve controls (black). These observations are reflected in the change in NND and RI. Data are presented as means±S.E.

We next analysed the pattern of RGC loss in the pONT model and its progression over time (Figure 3a). A significant loss of RGC density occurs rapidly from 3 days after model induction which plateaus by 56 days (*P*<0.001; Table 1). This effect is mirrored by a delayed but significant increase in RGC NND (Figure 3b) from baseline to 7 days post pONT induction (14.42 ±0.08  *versus* 16.88±0.75 *μ*m, *P*<*0.05*). RI appears initially insensitive to pONT model induction (3 days post pONT) but as the model progresses, from 7 days onwards (2.55±0.09, *P*<0.01), RI values begin to decline (Figure 3c). By 21 days post pONT induction, RI approaches 1.9 which is indicative of a retinal mosaic comprising randomly distributed RGCs.^37^ Further segmentation of the pONT retinal whole-mounts into quadrants (Figures 3d–f) reveals that significantly greater loss of RGC density was observed in the superior quadrant (Figure 3d, *P*<0.001) at 21 and 56 days post model induction. This pattern of RGC loss was reflected in significant differences in NND (Figure 3e, *P*<0.01) and RI values (Figure 3f, *P*<0.01 and *P*<0.05 for 21 and 56 days, respectively). Further segmentation of superior and inferior retinal quadrants into 15 concentric rings suggests that RGC loss in both superior and inferior sectors progresses quickly after pONT model induction and is primarily concentrated in the central superior retina.

### RGC loss predominantly occurs along the superior–inferior axis in the OHT model

**Figure 4:**
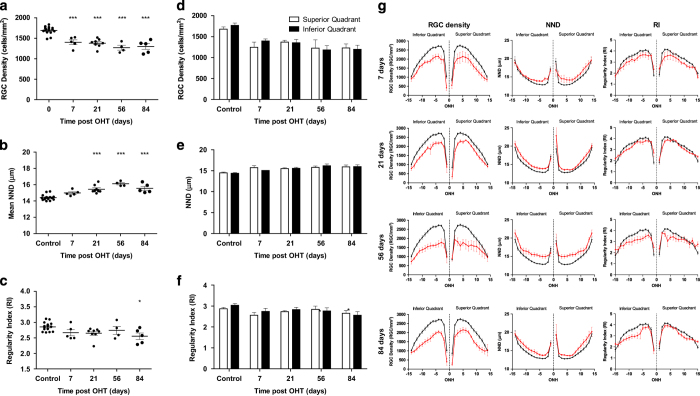
RGC survival after ocular hypertension (OHT) induction in the DA rat. Whole-retinal profiles of OHT induced loss of (**a**) RGC density, (**b**) nearest neighbour distance and (**c**) regularity index demonstrating significant reducing in RGC populations over the course of this model with only minor degradation in the regularity of the retinal mosaic (one-way ANOVA with Dunnets’ *post hoc* test *versus* naïve controls). (**d**–**f**) Comparison of RGC survival in the superior *versus* inferior retinal quadrants after ocular hypertension (OHT) induction in the DA rat. Profile of OHT induced loss of (**d**) RGC density, (**e**) nearest neighbour distance and (f) regularity index in the superior (white) and inferior (black) retinal quadrants as outlined in Figure 1. No significant differences in RGC loss were detected between the superior and inferior retinal segments (paired two-tailed *T*-test). (**g**) Further segmentation of RGC populations in the superior and inferior quadrants into a series of 15 non-overlapping concentric rings centred on the ONH suggest that a similar extent of RGC density loss occurs in the superior and inferior retinal quadrants over the course of this model (red) when compared with naïve controls (black). These observations are reflected in the change in NND and RI. Data are presented as means±S.E.

The decline in RGC density observed in the Morrison’s OHT model was less than that observed in the pONT model (Figure 4a and Table 1). IOP peaked 24h post OHT model induction with an average IOP of 19.9±3.2 *versus* 12.0±4.0 mm Hg in untreated contralateral eyes. A significant increase in IOP was observed in all OHT eyes (*P*<0.05) *versus* untreated contralateral eyes, similar to those previously reported by our group.^42^ A significant loss of RGC density was observed from 7 days post OHT induction (Figures 4a, *P*<0.001), which was accompanied by significant increases in NND (Figure 4b) from 21 days (14.42 ±0.08  *versus* 15.71 ±0.28 *μ*m for control and 21 days post OHT model induction). RI exhibited only a mild decline over the course of this model (Figure 4c), only achieving significance at 84 days post OHT induction (2.86±0.03 *versus* 2.55±0.11, *P*<0.05 for controls and 84 days post OHT model induction, respectively), suggesting retinal mosaic regularity was largely preserved, likely due to the relatively mild IOP elevation in this model.

Segmentation of the OHT retinal whole-mounts into quadrants (Figures 4d–f) reveals that RGC loss occurs at a similar rate throughout the entire retina, with no significant difference in RGC density (Figure 4d), NND (Figure 4e) or RI (Figure 4f) in superior and inferior quadrants at any time point investigated. Further segmentation of superior and inferior quadrants into 15 concentric rings reveals that RGC density declines more rapidly in the retinal centre than in the periphery (Figure 4g). This observation is mirrored by more pronounced increases in NND and declines in RI in the central retina compared with the retinal periphery over the course of this model.

### Significant RGC loss occurs in contralateral eyes in the OHT model

**Figure 5:**
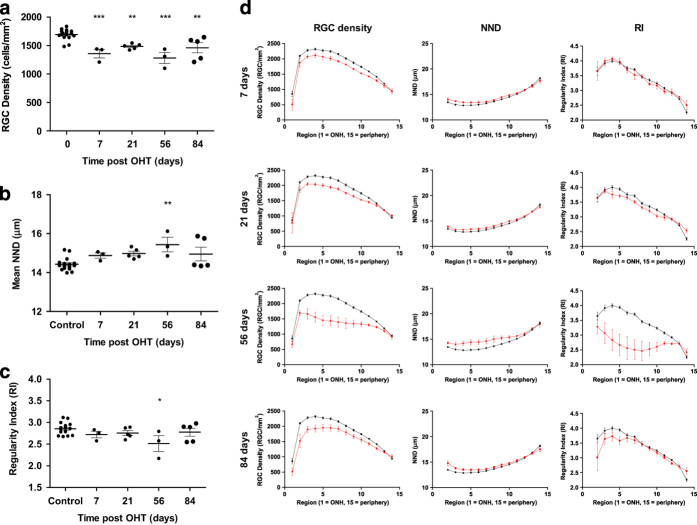
Retinal ganglion cell (RGC) survival after contralateral ocular hypertension (OHT) induction in the DA rat. Whole-retinal profile of (**a**) RGC density, (**b**) nearest neighbour distance (NND) and (**c**) regularity index demonstrating significant reducing in RGC populations when OHT was inducted in the opposite eye (Figure 4) over the course of this model with only minor degradation in the regularity of the retinal mosaic (one-way ANOVA with Dunnets’ *post hoc* test *versus* naïve controls). (**d**) Segmentation of RGC populations into a series of 15 non-overlapping concentric rings centred on the ONH suggest that greatest loss of RGC density occurs in the central *versus* the peripheral retina over the course of the OHT model (red) when compared with naïve controls (black). These observations are reflected in the change in NND and RI. Data are presented as means±S.E.

Analysis of contralateral untreated eyes from OHT cohorts was next performed (Figure 5). A significant decline in RGC density (Figure 5a) was observed from 7 days post OHT induction (*P*<0.001) (in an IOP related relationship), but to a lesser extent than eyes which were subject to elevated IOP (average RGC density loss 84 days post OHT induction 23±5% *versus* 14±6% for OHT and contralateral eyes, respectively, Table 1). Average NND increased from 7 days but only reached significance (*P*<0.01) *versus* naïve controls 56 days post OHT inductions (14.42±0.18 *versus* 15.44±0.37, *P*<0.01; Figure 5b). A similar trend was observed in RI (2.86±0.03 *versus* 2.51±0.18, *P*<0.05; Figure 5c), suggesting preservation of RGC retinal mosaic. Further segmentation of retinal whole-mounts into 15 concentric rings (Figure 5d) reveals that loss of RGC density and NND occurs primarily in the central retinal and that despite significant loss of RGCs, RI remains largely unaffected, indicating preservation of the retinal mosaic.

### Pattern of RGC loss reveals primary and secondary degenerative changes in OHT model comparable to human optical coherence tomography retinal nerve fibre layer segmentation

**Figure 6:**
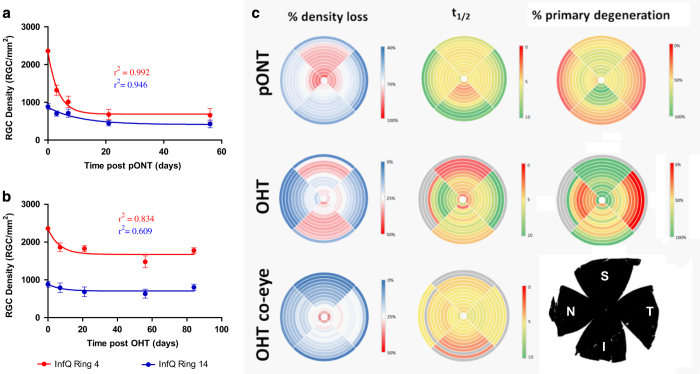
Summary of the rates of RGC loss in retinal segments of the pONT and OHT models. Example profiles of RGC density loss in the central (Ring 4, red) and peripheral (Ring 14, blue) inferior retina form the (**a**) pONT and (**b**) OHT models fit to a single-phase exponential decay model (equation (3)). (**c**) These data were used to construct colour maps summarising the percentage of RGC density loss over the course of each model, the half-life of RGC loss in each retinal sector on fitting to a single exponential decay model (equation (3)) and estimate the percentage of primary degeneration on fitting to a two-phase decay model (equations (4), (5), (6) as described in the text.

Clinically, optical coherence tomography retinal nerve fibre layer (RNFL) thickness changes are assessed by quadrant over time.^43,44^ We next investigated whether quadrant analysis could be used to define the rate of RGC density loss. Mean RGC density in each quadrant ring was plotted over time (Figures 6a and b) and fit to exponential decay equations (equations (4), (5), (6)). Results were used to construct colour maps of percentage RGC density loss and half-life and to estimate the percentage of primary degeneration in each ring segment (Figure 6c). Figure 6c illustrates that in the pONT model, the greatest percentage of RGC density loss occurred in the superior and central retinal segments (red regions) and least in the inferior segment and retinal periphery (blue sectors). This observation broadly correlated with half-life observations which were generally lower in the superior and central sectors (*t*_1/2_~4 days, yellow sectors) *versus* the inferior periphery sectors (*t*_1/2_>10 days, green sectors). Interestingly the lowest half-life of RGC density decay was observed in the central inferior retina (*t*_1/2_~9 days, orange sectors). By comparison, in the OHT model, the greatest percentage loss of RGC density was observed in the superior and inferior retinal sectors (~30% RGC density loss, red), *versus* the nasal and temporal quadrants (20–0%, white–blue sectors). Half-lives of RGC decay in this model were less in the superior retina than in other sectors, (average *t*_1/2_ superior retina=2.5 *versus* 4.7, 9.2 and 7.4 days in the inferior, nasal and temporal retina, respectively). This pattern of RGC loss is in agreement with previous observations using axonal sections and optical coherence tomography in the same rodent model of OHT, where greater RNFL thinning was observed in the superior–inferior axis *versus* the nasal–temporal axis.^25,42^ RNFL thinning has been suggested as a risk factor for future visual field loss.^45^ The pattern of RGC loss observed also closely matches the bowtie-like pattern of RNFL loss previously reported in human glaucoma patients^46^ and is supported by recent clinical observations where significant thinning of the RNFL in the superior and inferior retina was observed in a population of glaucoma suspects.^47^

Finally, to estimate the percentage of primary and secondary degeneration in each retinal sector, RGC density decay curves were fit to a two-phase exponential decay model, fixing rates of RGC loss at *t*_1/2_=1.7 and 16.3 days for primary and secondary RGC degeneration as reported previously.^48^ Figure 6c illustrates that in the pONT model of retinal degeneration, a combination of primary and secondary degeneration was observed in the superior and central retina (65% primary degeneration, green sectors) and the proportion of primary degeneration declined on approaching the superior retinal periphery (46% primary degeneration, yellow sectors). Surprisingly, the greatest occurrence of primary degeneration was observed in the central inferior retina, peaking at 84% in ring 4, while the lowest incidence of primary degeneration was observed in the interior, nasal and temporal retinal periphery (20–0%, orange sectors).

## Discussion

This study describes a method for segmentation of Brn3a-labelled retinal whole-mounts to extract greater information regarding the spatial distribution and rate of RGC loss in the pONT and OHT models of retinal neurodegeneration than has previously been achieved (Figure 1). Automated analysis of retinal whole-mounts is preferable to manual counting of retinal sectors, as it minimises sampling error, unintentional bias and inter-operator variability. Using spatial and temporal analysis of RGC loss in these models, we reveal distinctive patterns of primary and secondary degeneration that correlate with previous observations and RNFL changes in clinical glaucoma. Finally, significant RGC loss was reported in the contralateral eyes of the OHT model, lending support to the hypothesis that systemic effects can exacerbate secondary RGC degeneration in clinical glaucoma.^49^

Using the algorithm described, naïve retina were found to possess a comparable number of RGCs and global RGC density as in previous studies using similar rat species.^50,51^ Over the course of the pONT model, RGC density loss was most pronounced in the superior retina in agreement with previous studies.^7,31,48,52^ However, in the present study, a significant reduction in RGC density was also observed in the inferior retinal segment as soon as 3 days post pONT induction, which is much earlier than the 28 days previously reported by Levkovitch-Verbin *et al.* (Figure 3). An explanation for this observation is that the Levkovitch-Verbin study utilised the retrograde Fluorogold labelling rather than Brn3a, which has previously been reported to provide an earlier indicator of RGC loss in axonal injury models.^50^ The observation that RGC numbers decline rapidly in all regions of the retina after pONT is in agreement with several recent studies, which report the rapid spread of oxidative and excitotoxic stress beyond the initial site of injury and mitochondrial changes within 24h of model induction.^53–55^ RGC density loss in both models was found to fit best to an exponential decay model, in common with previous studies of rodent ON crush and transection models.^52,56^

The algorithm uses concentric rings to divide retinal whole-mounts into a series of 60 non-overlapping sectors, from which the regional spatial and temporal pattern of RGC loss can be evaluated. The advantage of this method of analysis, compared with using a series of concentric circles, is that changes in the central retina can be effectively differentiated from peripheral retinal effects, an approach that has previously been used to eliminate point-pattern bias from Ripley’s K-function.^57^

Analysis of regional RGC loss revealed that RGC density declined more substantially in the central retinal sectors than the peripheral retina in the pONT model (Figures 3 and 6) is in agreement with previous observations using axotomy models.^29^ By comparison, RGC loss in the OHT model was milder than that observed for the pONT model, reflecting the duration (3 weeks) and magnitude of IOP elevation of the OHT model, in agreement with previous studies.^42^ RGC loss in this model was concentrated in the central retina (visual streak). The observation of central retinal defects in both models of retinal degeneration is in broad agreement with previous studies using rodent models of OHT and axonal injury.^29,58^ This is of particular interest, as it is increasingly recognised that patients with glaucoma present with more complex retinal-wide visual defects than solely loss of peripheral vision,^59^ and coincides with a growing body of evidence suggesting that early clinical glaucomatous damage occurs in the macular, the site of greatest RGC density in humans.^60^

Secondary degeneration refers to the degeneration of RGCs in response to any consequence of the primary insult, such as oxidative stress or release of intracellular glutamates reserves, which can occur rapidly after onset of injury.^1,33,61^ An advantage of the pONT model of RGC degeneration is that primary and secondary degeneration are considered spatially segregated, with approximately 75% of RGC loss in the superior retina but only 25% of RGC loss in the inferior retina reported to be a result of primary degeneration.^19^ In the present study, the half-life of RGCs in the pONT model was substantially less in superior and central retinal sectors *versus* the inferior, nasal and temporal retinal periphery, suggesting greater primary degeneration indeed occurs in these regions. A combination of primary and secondary degeneration was found to occur in both superior and inferior sectors of the pONT model in broad agreement with previous observations.^19^ However the observation that the central retina showed greater proportion of RGC loss than the peripheral retina in this model was unexpected and may be because the DA rat exhibit less ectopic axonal organisation than other species.^31^ By comparison, reductions in RGC density in the OHT model were mirrored by significant increases in mean NND. The change in distribution of normalised NNDs suggested a similar combination of clustered and diffuse RGC loss as observed in the pONT model, albeit to a lesser extent, reflecting the less severe insult. To the authors’ knowledge, this study presents the first evidence to suggest primary and secondary degenerative processes in the rat OHT model.

Contralateral eyes are frequently used as untreated controls in animal models of glaucoma. This study reports that eyes contralateral to those subject to short-term IOP elevation were found to undergo significant RGC loss *versus* naïve eyes, despite exhibiting no significant change in IOP (Table 1 and Figure 5). RGC density loss in collateral eyes was less than that observed in eyes subject to increased IOP, but nevertheless significant compared with controls (Table 1). RGC density loss was most pronounced in the central retina (Figure 5). The pattern of RGC loss was different to that observed in OHT eyes, in that the pattern was less well defined with greatest loss in the nasal–temporal sectors (Figure 6). The surprising observation of RGC loss in contralateral eyes of rat glaucoma models is not without precedence. For example, unilateral optic nerve injury in the rat has previously been associated with bilateral activation of the pro-apoptotic transcription factor c-jun in RGCs, microglial activation and glia proliferation,^62,63^ and upregulation of stress response factor βB2-crystallin;^64^ thought to play a role in neuroprotection and axonal regeneration.^65^ In murine models, contralateral upregulation of glial fibrillary acid protein, major histocompatibility complex-II (MHC-II) and the glial gap junction protein connexion 43 are reported, in addition to RGC loss.^66–68^ Several explanations have been proposed to describe the origin of the contralateral RGC degeneration observed in glaucoma models, including secretion of pro-inflammatory cytokines into the circulation, the propagation of degenerative signals from insulted to contralateral eyes via the visual centres of the brain or as a systemic autoimmune response.^69^ The present study supports a growing body of evidence to suggest contralateral RGC loss in rodent models of retinal degeneration, and presents data to suggest that different patterns of RGC loss in OHT and contralateral eyes are observed in the well-established rat OHT model of glaucoma.

In conclusion, this article describes a novel algorithm to automatically extract information to a greater granularity than has previously been achieved from Brn3a-labelled retinal whole-mounts. We show how this technique can be successfully used to investigate the extent and spatial segregation of primary and secondary degenerative processes in models of neurodegeneration. Furthermore, we demonstrate contralateral effects of OHT on RGC populations.

## Materials and Methods

### Animals

Adult male Dark Agouti rats (150–200 g) were treated with procedures approved by the UK Home Office and in compliance with the ARVO Statement for the Use of Animals in Ophthalmic and Vision Research. All animals (80 rats, 100 eyes) were maintained in a 12 h light (140–260 lux)–12h dark cycle, with food and water *ad libitum*.

### pONT model induction

pONT was performed in the left eye of 35 DA rats, using an adaptation of a previously described technique.^7,10^ Briefly, under general anaesthesia, an incision was made in the superior conjunctiva, exposing the optic nerve sheath. The optic nerve was then exposed using a longitudinal slit in the dura mater to allow a 0.2 mm cut to be made in the dorsal optic nerve 2 mm behind the eye using an ophthalmic scalpel with a steel cutting guard. Damage to major ophthalmic blood vessels was avoided and verified at the end by ophthalmoscopy. Animals were killed 3, 7, 21 and 56 days post pONT surgery. A population of bilaterally untreated (naïve) retinas were used as controls.

### OHT model induction

OHT was surgically induced in the left eye of 25 DA rats as described previously.^42^ IOP was elevated in the left eye of each animal by the injection of 0.05 ml hypertonic saline solution (1.80 M) into the two episcleral veins using a syringe pump (0.05 ml/min; UMP2; World Precision Instruments, Sarasota, FL, USA). A propylene ring, with a 1-mm gap cut out of its circumference, was placed around the equator to prevent injected saline outflow from other aqueous veins.

The IOP of both eyes in each rat was measured at regular intervals with a tonometer (TonoLab; Tiolat Oy, Heisinki, Finland) under inhalational anaesthesia (0.4% isoflurane in oxygen). For each animal, cumulative IOP exposure, defined as the integral of IOP elevation over time (mm Hg/day), was calculated from the area under the curve, as previously described. Animals were killed 7, 21, 56 and 84 days post-surgery. A population of bilaterally untreated (naïve) retinas were used as controls.

### Brn3a immunohistochemistry and confocal microscopy

After animals were killed, both eyes were enucleated and fixed in 4% paraformaldehyde at 4 °C overnight before dissecting retinal whole-mounts. Whole-mounts were stained for the RGC specific nuclear-localised transcription factor Brn3a using an anti-mouse mAb (1:500; Merk Millipore, Darmstadt, Germany) and examined under confocal microscopy (LSM 710; Carl Zeiss MicroImaging GmbH, Jena, Germany). Each retinal whole-mount was imaged as a tiled z-stack at ×10 magnification, which was used to generate a single plane maximum projection of the RGC layer in each retina for subsequent analysis (Figure 1a). Each whole-mount image was manually orientated so that the superior retina was towards the top of the image using *in vivo* cSLO imaging of retinal vasculature as a reference. Retina image acquisition settings were kept constant for all retinas imaged, allowing comparison of Brn3a expression in each experimental group as previously described.^50^

### Automated quantification of Brn3a-labelled RGCs in retinal whole-mounts

Quantification of Brn3a-labelled RGCs in retinal whole-mounts was achieved using an algorithm previously described.^10^ Briefly, a high-pass filter was first applied to the 8-bit Brn3a-labelled channel to remove background followed by application of a 130 intensity threshold. The ImageJ watershed algorithm was then used to separate touching particles and those within 7–21 *μ*m size range were counted based on RGC sizes previously reported.^34^ Only particles with a circularity >0.7 were considered to be RGCs. Once identified, a region of interest was defined for each RGC (Figure 1c) from which RGC area, mean grey pixel intensity and centroid (*x,y*) were recorded. The NND was determined for each RGC using an ImageJ macro developed by Yuxiong Mao (https://icme.hpc.msstate.edu/mediawiki/index.php/Nearest_Neighbor_Distances_Calculation_with_ImageJ) from which RI was calculated as described previously (equation (1)), where *x* is the mean nearest neighbour distance and *σ* is the standard deviation of nearest neighbour distance.^70^

(1)RI=x¯NNDσNND

### Whole-mount retinal area determination and segmentation

The area of each retina was determined by manually applying a low intensity threshold (0–5) to each image to create a mask of retinal whole-mount area (Figure 1b). Retinal area (black pixels) was measured using ImageJ as described previously.^10^ To determine the area of segmented retina, a series of 15 concentric rings of increasing radii (0.3 mm) centred on the ONH were applied to the whole-retinal mask and the areas of each subdomain were determined (Figure 1c). Rings were further subdivided into quadrants (Figure 1d) using the centre of the ONH (*a,b*) as the retinal centre in each case.

### Automated Brn3a-labelled RGC retinal segmentation

Once identified, RGCs were further segmented into a series of 15 concentric rings of increasing radii (0.6 mm) centred on (*a,b*). This was achieved by calculating the Euclidean distance of each RGC centroid relative to the ONH centre using equation (2), where (*x,y*) is the centroid of each RGC.

(2)d=(x−a)2+(y−b)2

For whole-ring retinal analysis the Euclidean distance [*D*] of each RGC was used to gate cells into a series of 15 concentric rings 0.6 mm in radii centred on the ONH. Rings were labelled 1 to 15 with ring 1 centred on the ONH and 15 on the retinal peripheral (Figure 1c). For quadrant analysis (Figures 1d and e), RGCs were gated into one of four quadrants: Temporal, Superior, Inferior and Nasal using Cartesian or polar coordinate based segmentation which gave identical results.

Mean RGC density was determined for each ring or by dividing the RGC count in each sector by the corresponding retinal area. Mean NND for each region was calculated by averaging NNDs for RGCs within each sector. In each case RI was calculated from mean NND and *σ*_NND_ of each sector, as described above. Colour mapped scatterplots were drawn for qualitative analysis using RGC centroid and NND values with Origin 2015 (OriginLabs, Northampton, MA, USA).

### Determining the rate of RGC primary and secondary degeneration

To determine half-life of RGC loss in both models, longitudinal profiles of RGC density in each retinal segment were fit to a one-phase exponential decay model with plateau (equation (3)) (Figure 6).

(3)y=(Y0-Plateau)×exp(-K×X)+Plateau

To determine the percentage of primary degeneration in each retinal segment, aforementioned RGC density profiles were fit to a two-phase exponential decay model with plateau (equations (4), (5), (6)). Results were presented as colour maps (Figure 6c),

(4)SF=(Y0−Plateau)×PF×0.01

(5)SS=(Y0-Plateau)×(100-PF)×0.01

(6)y=(Plateau+SF)×exp(-KF×X)+(Plateau+SS)×exp(-KS×X)

where *K*_F_ and *K*_s_ refer to the two rate constants fixed using the fast (primary degeneration) and slow (secondary degeneration) half-lives as described in the text, *S*_F_ and *S*_s_ refer to the span (distance between *Y*_0_ and Plateau) accounted by the fast and slow components of the equation, and *P*_F_ is the percentage of primary degeneration.

### Statistical analysis

All data were analysed with the Student’s *t*-test or one-way ANOVA with Dunetts *post hoc* test *versus* control groups using GraphPad Prism 5 (GraphPad Software, Inc., La Jolla, CA, USA), unless described otherwise. The longitudinal profiles of RGC degeneration following pONT and OHT induction were fitted with a one-phase and a two-phase exponential decay model with plateau as described in the text. Data were presented as means±S.E. and *P*<0.05 was considered significant.

## Figures and Tables

**Table 1:** Summary of pONT and OHT study treatment groups – mean (S.E.)

*Mean (S.E.)*	*Naïve controls*	*pONT model*				*OHT model*							
		*3 days*	*7 days*	*21 days*	*56 days*	*7 days OHT*	*7 days contralateral eye*	*21 days OHT*	*21 days contralateral eye*	*56 days OHT*	*56 days contralateral eye*	*84 days OHT*	*84 days contralateral eye*
*N* (starting *N*)	16 (20)	5 (10)	7 (10)	9 (10)	5 (5)	5 (5)	5 (5)	9 (10)	5 (5)	4 (5)	3 (5)	5 (5)	5 (5)
Mean RGC count	82 592 (681)	64 844 (2468)	47 512 (5357)	20 957 (2612)	25 596 (6606)	73 730 (4249)	81 274 (447)	67 061 (5045)	83 559 (1490)	61 058 (3562)	63 514 (4939)	76 851 (3598)	78 384 (1852)
Density (RGC/mm^2^)	1695 (23.3)	1233 (78.2)	894.5 (98.4)	404.3 (52.9)	491.7 (120.4)	1404 (58.1)	1359 (74.7)	1303 (64.9)	1485 (21.9)	1271 (59.5)	1283 (98.2)	1298 (68.4)	1462 (89.7)
RGC density loss (% of controls)	—	27 (2)	47 (5)	76 (10)	71 (17)	17 (1)	20 (1)	23 (5)	12 (2)	25 (5)	24 (8)	23 (5)	14 (6)
*P-*value	—	***	***	***	***	***	***	***	**	***	***	***	**
Peak IOP (mm Hg)	9.9 (0.1)	—	—	—	—	19.6 (0.8)	11.2 (0.7)	20.6 (1.3)	10.4 (0.6)	20.9 (2.7)	11.6 (1.1)	18.0 (0.9)	10.0 (0.4)

***P<0.001, one-way ANOVA with Dunnetts *post hoc* test *versus* control condition RGC density. Starting *N* includes retina lost due to tearing during dissection and whole-mounting process.
